# Bacterial Engulfment Mechanism Is Strongly Conserved in Evolution Between Earthworm and Human Immune Cells

**DOI:** 10.3389/fimmu.2021.733541

**Published:** 2021-09-01

**Authors:** Bohdana Kokhanyuk, Kornélia Bodó, György Sétáló Jr, Péter Németh, Péter Engelmann

**Affiliations:** ^1^Department of Immunology and Biotechnology, Clinical Center, Medical School, University of Pécs, Pécs, Hungary; ^2^Department of Medical Biology and Central Electron Microscope Laboratory, Medical School, University of Pécs, Pécs, Hungary; ^3^Signal Transduction Research Group, János Szentágothai Research Centre, Pécs, Hungary

**Keywords:** macrophages, coelomocytes, evolution, endocytosis, phagocytosis, inhibitors

## Abstract

Invertebrates, including earthworms, are applied to study the evolutionarily conserved cellular immune processes. Earthworm immunocytes (so-called coelomocytes) are functionally similar to vertebrate myeloid cells and form the first line of defense against invading pathogens. Hereby, we compared the engulfment mechanisms of THP-1 human monocytic cells, differentiated THP-1 (macrophage-like) cells, and *Eisenia andrei* coelomocytes towards *Escherichia coli* and *Staphylococcus aureus* bacteria applying various endocytosis inhibitors [amantadine, 5-(*N*-ethyl-*N*-isopropyl) amiloride, colchicine, cytochalasin B, cytochalasin D, methyl-ß-cyclodextrin, and nystatin]. Subsequently, we investigated the messenger RNA (mRNA) expressions of immune receptor-related molecules (*TLR*, *MyD88*, *BPI*) and the colocalization of lysosomes with engulfed bacteria following uptake inhibition in every cell type. Actin depolymerization by cytochalasin B and D has strongly inhibited the endocytosis of both bacterial strains in the studied cell types, suggesting the conserved role of actin-dependent phagocytosis. Decreased numbers of colocalized lysosomes/bacteria supported these findings. In THP-1 cells *TLR* expression was increased upon cytochalasin D pretreatment, while this inhibitor caused a dropped *LBP/BPI* expression in differentiated THP-1 cells and coelomocytes. The obtained data reveal further insights into the evolution of phagocytes in eukaryotes. Earthworm and human phagocytes possess analogous mechanisms for bacterial internalization.

## 1 Introduction

The cellular uptake mechanism of macromolecules, nutrients, and various particles is generally considered as endocytosis. Its action is strongly related to the plasma membrane, which, by invaginating, forms vesicles containing transported molecules. Endocytic pathways govern essential functions such as cell–cell communication, signal transduction, immune response, and cellular homeostasis. Generally, two major types of endocytosis can be distinguished in the cells: phagocytosis and pinocytosis (1). Phagocytosis is carried out against particles larger than >0.5 μm by specialized cells to protect the organism from microbes and eliminate damaged cells (2).

At least four types of pinocytosis mechanisms exist in the cells, among which are macropinocytosis, clathrin-mediated endocytosis (CME), caveolae-mediated endocytosis (CvME), and clathrin/caveolin-independent endocytosis. All these pathways differ from the point of the origin of the particle engulfed, size, and mechanism of endocytic vesicle formation (1).

Due to its importance in cellular homeostasis, endocytosis (phagocytosis and pinocytosis) is a phylogenetically conserved trafficking process that evolved from amoeba to human cells (3). It seems that phagocytosis is lost from fungi and plants. Interestingly, it is suggested that the primary forms of uptake mechanisms coevolved with nutrition intake and only later opted as immune/defense mechanism during metazoan phylogenesis (4). To eliminate pathogen structures, distinct macrophage types (e.g., archeocytes, hemocytes, coelomocytes, microglia, Kupffer cells) have been developed in the diverse animal groups (5).

In this regard, well-conserved cellular and humoral innate immune components have emerged in invertebrates to keep their self-integrity similarly to those in vertebrates. For instance, segmented worms (earthworms) are “up and coming” research animal models to study the phylogenesis of immunity (6, 7). Earthworms lack adaptive immunity that allows to solely study the mechanisms of innate immunity. Earthworm’s body cavity (coelomic cavity) is an immunologically competent compartment containing motile immune cells (so-called coelomocytes) and a fluid abundant in multifunctional proteins (coelomic fluid) (7). Coelomocytes can be subdivided into two major subpopulations (amoebocytes and eleocytes). These cells possess a variety of immune functions that resembles vertebrate leukocytes. In particular, amoebocytes are capable of phagocytosis and encapsulation, while eleocytes produce antimicrobial factors and maintain homeostasis (8).

To this end, we hypothesized that earthworm amoebocytes resemble vertebrate myeloid lineage, not only on functional but also molecular levels. To test this theory, applying various endocytosis inhibitors, we compared the bacterial uptake mechanisms, intracellular localization of engulfed bacteria, and mRNA expression of pattern recognition receptors (PRRs) between earthworm coelomocytes and the vertebrate counterpart (THP-1 human monocytic cell line).

## 2 Materials and Methods

### 2.1 Cell Culture Conditions

Human monocytic leukemia cell line THP-1 (ATCC^®^ TIB-202™) was cultured in Roswell Park Memorial Institute (RPMI) medium supplemented with 10% heat-inactivated fetal bovine serum (FBS, Euroclone, Milan, Italy) and 1% penicillin/streptomycin (100 U/ml penicillin and 100 µg/ml streptomycin, Lonza, Basel, Switzerland) at 37°C in a humidified 5% CO_2_ atmosphere. THP-1 cells were differentiated (diff. THP-1 cells) using 5 ng/ml phorbol 12-myristate-13-acetate (PMA) for 48 h applying the aforementioned culture conditions to obtain macrophage-like cells (9). Differentiation of monocytes was verified with light microscopy.

### 2.2 Earthworm Husbandry and Coelomocyte Isolation

Adult *Eisenia andrei* (Oligochaeta, Lumbricidae) were maintained at standard laboratory conditions at room temperature (21°C) and fed with manure soil (10). Before coelomocyte isolation, earthworms were placed overnight onto moist paper towel to empty their digestive tract. Coelomocytes were collected by applying an extrusion buffer and washed in *Lumbricus* balanced salt solution (LBSS) as described earlier (10). Density of coelomocytes was enumerated (1 × 10^6^ cells/ml in each assay) by a dead-cell exclusion method applying trypan blue dye.

### 2.3 Endocytosis Inhibitor Treatments

THP-1 cells, diff. THP-1 cells, and coelomocytes were placed onto 24-well plates and pretreated with different pharmacological pathway inhibitors: cytochalasin D, cytochalasin B, colchicine, 5-(*N*-ethyl-*N*-isopropyl) amiloride (EIPA), amantadine, methyl-ß-cyclodextrin, and nystatin. All inhibitors were purchased from Sigma-Aldrich (St. Louis, MO, USA). Applied inhibitor concentrations and incubation time were selected based on the previous literature [(11–17), please see **Table 1**]. For energy-dependent uptake inhibition, the target cells were preincubated at 4°C for 30 min to block any active uptake mechanism (18). Prior to flow cytometry measurements, samples were stained with 7-aminoactinomycin D (7-AAD, 1 µg/ml, Biotium, Fremont, CA, USA) for the detection of cell viability.

**Table 1:** List of the pharmacological inhibitors and their applied conditions in the endocytosis experiments.

Inhibitor	Target pathway	Mode of action	Applied conditions	References
Cytochalasin B	Phagocytosis	F-actin depolymerization	5 µM, 1 h	(11)
Cytochalasin D			5 µM, 1 h	(12)
Colchicine	Pinocytosis	Inhibits microtubule polymerization	100 µM, 2 h	(13)
5-(*N*-ethyl-*N*-isopropyl) amiloride (EIPA)	Macropinocytosis	Blocking the Na^+^/H^+^ exchanger	5 µM, 30 min	(14)
Amantadine	CME	Blocking the budding of clathrin-coated pits	500 µM, 30 min	(15)
Methyl-ß-cyclodextrin (MßCD)	CvME	Removing cholesterol out of the plasma membrane	1 mM, 30 min	(16)
Nystatin	CvME	Cholesterol sequestration	54 µM, 30 min	(17)

### 2.4 *In Vitro* Bacterial Challenge

Initially, THP-1 cells, diff. THP-1 cells, and coelomocytes were incubated with fluorescein isothiocyanate (FITC)-coupled, heat-inactivated *Escherichia coli* (ATCC 25922) and *Staphylococcus aureus* (OKI 112001) for 1, 2, 4, 16, and 24 h (THP-1 and diff. THP-1 at 37°C) or 1, 2, 4, 16, 24, and 48 h (coelomocytes at room temperature) to assess bacterial uptake kinetics. Immune cells (10^6^) and bacteria cells (10^7^) were incubated together in 1 ml of cell culture media (8). Prior to flow cytometry, trypan blue was added to differentiate bound/ingested bacteria by quenching. For uptake inhibition analysis, inhibitor-treated target cells were incubated with bacterial strains at appropriate temperatures (except during energy-dependent uptake inhibition target cells were kept at 4°C) for 24 h. Besides, vehicle controls were also applied. Samples have been washed and resuspended in phosphate-buffered saline (PBS) (THP-1 cells and diff. THP-1 cells) or LBSS (coelomocytes). Fluorescence signals were measured in FL1 (530/30 filter) gated on living cells, as detected by 7-AAD in FL3 (670 LP filter).

### 2.5 Flow Cytometry

Flow cytometry analysis was carried out with a FACSCalibur flow cytometer (Beckton Dickinson, Franklin Lakes, NJ, USA). During experiments, 30,000 events per sample were measured based on their forward scatter (FSC) and sideward scatter (SSC) characteristics. Results were analyzed with FCS Express software (De Novo Software, Glendale, CA, USA).

### 2.6 Labeling F-Actin With Phalloidin and Lysosome Staining

After inhibitor treatment and incubation with FITC-coupled bacterial strains, cells (80 µl of 1 × 10^5^/ml) were spread onto slides applying Cytospin 3 (SHANDON, Thermo Scientific, Waltham, MA, USA). Cells were fixed in 4% paraformaldehyde, washed, and permeabilized with PBS/0.1% Triton X-100 with 5% bovine serum albumin (BSA) for 20 min. F-Actin was stained with CF568 Phalloidin (Biotium, Fremont, CA, USA) or Alexa Fluor 488 Phalloidin (Invitrogen Molecular Probes, Eugene, OR, USA) for 45 min. For lysosome staining, samples were incubated with LysoTracker^®^ Red DND-99 (Molecular Probes, Eugene, OR, USA) at a final concentration of 100 nM for 30 min, room temperature, in the dark followed by nuclear counterstaining with 4′,6-diamidino-2-phenylindole (DAPI, Sigma-Aldrich).

### 2.7 Confocal Laser Scanning Microscopy and Quantitative Colocalization Analysis

Confocal laser scanning microscopy (CLSM) images were generated using an Olympus FV-1000 laser scanning system. Single optical sections were taken with a 40×, long working distance phase objective at zoom settings 3, 4, 5, or 6 for fluorescent captures. For immersion images, the magnification was 60×. Images were then processed and merged with ImageJ1.x software.

CLSM images were used to quantify the colocalization of FITC-conjugated *E. coli* and *S. aureus* with LysoTracker labeled lysosomes. At least 14 lysosomes were evaluated from each condition. The Coloc2 plug-in for Fiji was used to determine the Manders’ coefficient after applying region of interest (ROI) for each microscopic picture (19, 20). Manders’ coefficient measures the fraction of one dye (green) that colocalizes with a second dye (red); M1 measures the fraction of bacteria colocalizing with lysosomes.

### 2.8 Statistical Analysis

All the experiments were performed at least three independent times. Results were analyzed with GraphPad Prism 8.0 software (La Jolla, CA, USA). Data were expressed as mean ± SD of the values received in independent experiments. One-way ANOVA or two-way ANOVA with multiple comparison *post-hoc* test was carried, out and the significance of data was evaluated. Differences were considered significant if *p* < 0.05.

## 3 Results

### 3.1 Actin Polymerization Inhibitors Attenuate the Bacterial Engulfment by THP-1 Cells

**Figure 1:**
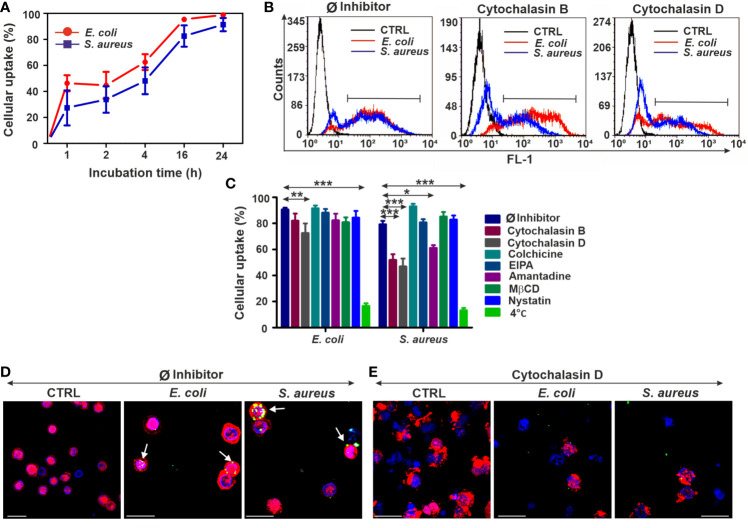
The upshots of endocytosis inhibition on the bacterial uptake in THP-1 cells. **(A)** Kinetics of FITC-*E. coli* and FITC-*S. aureus* bacteria uptake by THP-1 cells after 1, 2, 4, 16, and 24 h of incubation. **(B)** Representative flow cytometry histograms of THP-1 cells after 24 h of bacteria engulfment without or with inhibitors (5 µM cytochalasin B/cytochalasin D). **(C)** Efficiency of different endocytosis inhibitors on the bacterial uptake of THP-1 cells. The results are presented as mean ± SD of four replicates. Asterisks denote statistical significance (*p < 0.05, **p < 0.01, ***p < 0.001) between the Ø inhibitor control and different treatments. **(D)** Merged CLSM images of THP-1 cells incubated in the absence of inhibitors and **(E)** in the presence of 5 µM cytochalasin D that demonstrate the changes in the actin cytoskeleton (red, CF568 Phalloidin) and inhibition of bacterial (green, FITC-*E. coli* and FITC-*S. aureus*) uptake. Nuclear counterstaining was performed with DAPI (blue). Note the overlapping signal between engulfed bacteria and actin filaments (arrows, **D**). Scale bars: 50 µm.

First, THP-1 cells were exposed to FITC-coupled heat-inactivated *E. coli* and *S. aureus* at different time points to estimate their uptake capacity. Kinetic analysis revealed the prompt bacteria engulfment starting from the first hour of incubation and reaching approximately 90% of cellular uptake at 24 h in THP-1 cells (**Figure 1A**). Next, prior to adding bacteria, we pretreated THP-1 cells with different endocytosis pathway inhibitors (**Table 1**) to obtain information about the uptake mechanisms. Cytochalasin D and B were applied to inhibit phagocytosis, colchicine for pinocytosis blocking, EIPA to disrupt macropinocytosis, amantadine to inhibit CME and MßCD, and nystatin to block CvME.

Among all applied inhibitors, cytochalasin B significantly inhibited only the engulfment of *S. aureus* (**Figures 1B, C**), regardless of its weak toxicity against THP-1 cells (**Supplementary Figure 1A**). In contrast, cytochalasin D significantly blocked the uptake of both bacterial strains (**Figures 1B, C**) compared to Ø inhibitor controls (**Figures 1B, C**). There is also a slight inhibitory effect of amantadine for *S. aureus* engulfment (**Figure 1C**), while other tested inhibitors did not reduce significantly the bacterial uptake in THP-1 cells (**Figure 1C**). The highest inhibition rate in bacterial engulfment was detected when THP-1 cells were incubated with bacteria at 4°C to assess the energy dependence of endocytosis (**Figure 1C**).

CLSM studies using phalloidin showed colocalization of engulfed FITC-coupled bacteria and labeled actin, which supports the involvement of actin-dependent endocytosis in the bacterial uptake (**Figure 1D**). The preincubation of cells with cytochalasin D resulted in disruption of actin polymerization and visible change of its structure (**Supplementary Figure 2**) that subsequently leads to a biased internalization of bacteria (**Figure 1E**).

### 3.2 Differentiated THP-1 Cells Resemble Normal THP-1 Monocytes During the Bacterial Engulfment

**Figure 2:**
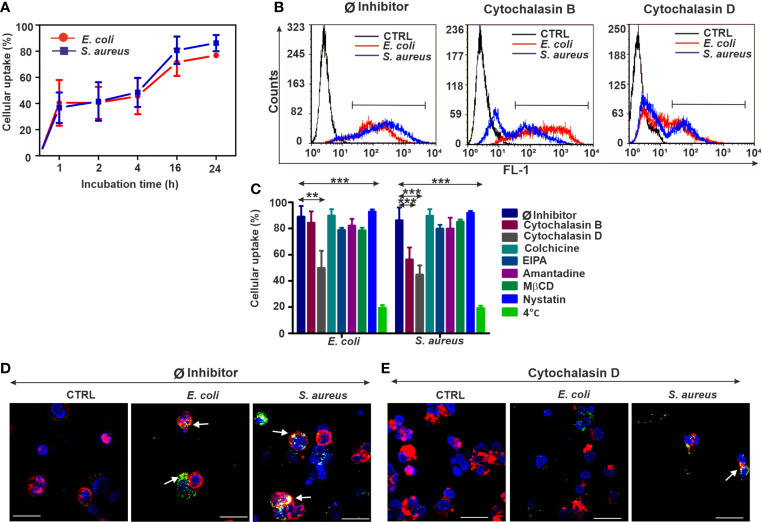
Biased bacterial engulfment in diff. THP-1 cells upon endocytosis inhibition. **(A)** Kinetics of FITC-*E. coli* and FITC-*S. aureus* uptake by diff. THP-1 cells after 1, 2, 4, 16, and 24 h of incubation. **(B)** Representative flow cytometry histograms of diff. THP-1 cells after 24 h of bacteria uptake, without or with inhibitors (5 µM cytochalasin B/cytochalasin D). **(C)** Efficiency of various endocytosis blockers on the bacterial engulfment in diff. THP-1 cells. The results are presented as mean ± SD of four replicates. Asterisks mark statistical significance (**p < 0.01, ***p < 0.001) between the Ø inhibitor control and various treatments. **(D)** Merged CLSM images of diff. THP-1 cells in the absence of endocytosis inhibitors or **(E)** in the presence of 5 µM cytochalasin D that demonstrate the changes in the actin cytoskeleton (red, CF568 Phalloidin) and inhibition of bacterial (green, FITC-*E. coli* and FITC-*S. aureus*) uptake. Nuclear counterstaining was performed with DAPI (blue). Note the overlapping signal between engulfed bacteria and actin filaments (arrows, **D**). Scale bars: 50 µm.

The macrophage-like phenotype of THP-1 cells (diff. THP-1) was achieved by applying a PMA stimulation for 48 h. Kinetic analysis of bacterial engulfment by diff. THP-1 cells (**Figure 2A**) revealed similar trends to those of normal THP-1 cells (**Figure 1A**). Until 24 h of incubation, *E. coli* internalization reached approximately 90% and somewhat less for *S. aureus* (**Figure 2A**). Subsequently, we analyzed the effects of various inhibitors on the bacterial uptake by diff. THP-1 cells (survival rate of diff. THP-1 cells was only affected by colchicine, **Supplementary Figure 1B**). While cytochalasin B only decreased the *S. aureus* engulfment rate, the strongest inhibition was evoked by cytochalasin D in diff. THP-1 cells (**Figures 2B, C**) and in normal THP-1. We did not observe any effects in the case of other inhibitors (**Figure 2C**). The energy dependence of bacterial uptake was monitored at 4°C and evidenced strong inhibition similarly to normal THP-1 cells (**Figure 2C**).

By means of CLSM, an extensive bacterial uptake in untreated diff. THP-1 cells was observed (**Figure 2D**). Imaging of the diff. THP-1 immunocytes exposed to cytochalasin D (**Figure 2E**, **Supplementary Figure 2**) has supported the previous results of bacterial uptake inhibition obtained by flow cytometry.

### 3.3 Coelomocyte-Mediated Bacterial Engulfment Is Blocked by Actin Depolymerization

According to physical parameters such as size and granularity, the two major coelomocyte subpopulations (e.g., amoebocytes, and eleocytes) can be distinguished by flow cytometry (8). In our uptake experiments, we have focused on amoebocytes, since this population is involved in the cellular antibacterial defense among coelomocyte subtypes (8). In addition, eleocytes possess a strong autofluorescence (derived from riboflavin) that hampers their fluorescence dye-based analysis (21).

**Figure 3:**
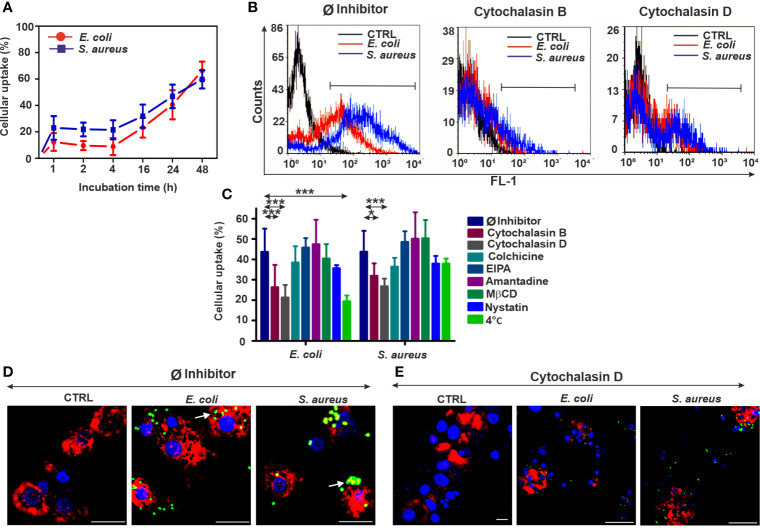
Reduced bacterial uptake in the coelomocytes following endocytosis inhibition. **(A)** Uptake kinetics of FITC-*E. coli* and FITC-*S. aureus* bacteria by coelomocytes after 1, 2, 4, 16, 24, and 48 h of incubation. **(B)** Representative flow cytometry histograms after 24 h of bacteria engulfment without or with inhibitors (5 µM cytochalasin B/cytochalasin D). **(C)** Efficiency of numerous endocytosis inhibitors on the bacterial uptake in coelomocytes. The results are exhibited as mean ± SD of four replicates. Asterisks indicate statistical significance (*p < 0.05, ***p < 0.001) between the Ø inhibitor control and different treatments. **(D)** Merged CLSM images of coelomocytes in the absence or **(E)** presence of 5 µM cytochalasin D that display the changes in the actin cytoskeleton (red: CF568 Phalloidin) and inhibition of bacterial (green, FITC-*E. coli* and FITC- *S. aureus*) uptake. Nuclear counterstaining was performed with DAPI (blue). Note the overlapping signal between engulfed bacteria and actin filaments (arrows, **D**). Scale bars: 20 µm.

In the case of coelomocytes (amoebocytes), the bacterial uptake starts to gradually increase at 4 h and reaches about 70% at the end of the observation period (48 h) (**Figure 3A**). Cytochalasin D showed slight cytotoxicity for earthworm coelomocytes (**Supplementary Figure 1C**); nevertheless, it has efficiently blocked their endocytic activity similarly to human immunocytes (**Figures 3B, C**). Interestingly, Gram-negative bacterial uptake was also blocked by cytochalasin B (**Figures 3B, C**) on the contrary to normal and diff. THP-1 cells (**Figures 1C**, **2C**). Similarly, to the human cells, other endocytosis inhibitors did not cause any noticeable blocking effects in coelomocytes; however, in the case of nystatin, we observed a non-significant trend of decreased engulfment rate (**Figure 3C**).

Inhibition of energy-dependent endocytosis (coelomocytes at 4°C) did not rescue *S. aureus* bacteria from engulfment. Even the blockade of *E. coli* internalization was less extended compared to the human counterparts (**Figure 3C**). CLSM analysis of phalloidin costained coelomocytes (Ø inhibitor control, **Figure 3D**) has revealed the immense uptake of target bacteria, especially in the case of *S. aureus*. In line with flow cytometry measurements, CLSM analysis has supported that cytochalasin D pretreatment efficiently impairs actin polymerization (**Supplementary Figure 2**) and bacterial uptake by coelomocytes (**Figure 3E**).

### 3.4 Decreased Colocalization of Lysosomes and Engulfed Bacteria by Actin Polymerization Blockers

**Figure 4:**
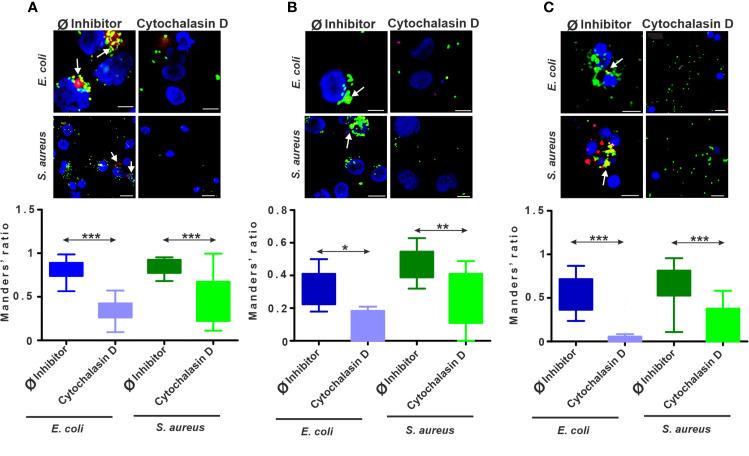
The intracellular colocalization of lysosomes and bacteria is biased upon endocytosis inhibition. Representative CLSM images and Manders’ colocalization coefficients ratios for **(A)** THP-1 cells, **(B)** diff. THP-1 cells, and **(C)** coelomocytes after 24 h incubation with bacteria in the absence or the presence of 5 µM cytochalasin D. Representative images demonstrate the colocalization of FITC-coupled bacteria (green) with the LysoTracker labeled (red) lysosomes (arrows). Nuclear counterstaining was performed with DAPI (blue). Scale bars: 10 µm; 50 µm (THP-1 with *S. aureus*). The corresponding graphs show the fraction of colocalized *E. coli*/lysosomes and *S. aureus*/lysosomes. Results are presented as mean ± SD from three independent experiments. Asterisks point out statistical significance (*p < 0.05, **p < 0.01, ***p < 0.001) between the Ø inhibitor control and cytochalasin D exposed cells.

To track the intracellular fate of fluorescent bacterial particles, earthworm and human immune cells were stained with LysoTracker Red (**Figure 4** and **Supplementary Figures 3–5**). In the course of CLSM analysis, we observed stronger fluorescent signals from lysosomes in bacteria-treated cells compared to the unexposed, control THP-1 cells (**Supplementary Figure 3A**). Application of cytochalasin D has lowered the bacterial uptake by THP-1 cells; hence, it significantly decreased the amount of colocalized signal (**Supplementary Figure 3B**), which was statistically confirmed by Manders’ colocalization coefficient value analysis (**Figure 4A**). The fraction of colocalized *E. coli*/lysosomes and *S. aureus*/lysosomes upon the cytochalasin D treatment was nearly two times lower compared to the Ø inhibitor control values (**Figure 4A**).

Similarly, to normal THP-1 cells, in diff. THP-1 cells, the Manders’ coefficient analysis revealed that the fluorescent signals from colocalized bacteria and lysosomes significantly decreased upon cytochalasin D treatment (**Figure 4B** and **Supplementary Figures 4A, B**).

Regarding the role of lysosomes in the elimination of ingested bacteria, the uptake inhibition by cytochalasin D was demonstrated by CLSM in coelomocytes (**Figure 4C**, **Supplementary Figures 5A, B**). We observed more than 10-fold decrease in colocalized *E. coli*/lysosomes and more than 3-fold decrease in colocalized *S. aureus*/lysosomes (**Figure 4C**) in the case of coelomocytes.

### 3.5 Pattern Recognition Receptor mRNA Levels Vary Upon Cytochalasins Exposure

Elimination of microbial pathogens is facilitated by evolutionarily conserved pattern recognition receptors (PRRs). Certain homologs of PRRs have been described from earthworms (22). To assess their possible involvement during the endocytosis in earthworm and human cells, we tested the mRNA expression levels of *toll-like receptor (TLR)*, *myeloid differentiation factor 88 (MyD88)*, and *lipopolysaccharide-binding protein/bactericidal permeability-increasing protein (LBP/BPI)* [for detailed methodological description of RNA isolation, complementary DNA (cDNA) synthesis, and the conditions of qPCR measurements, please see the **Supplementary Material**] upon bacterial challenge with or without endocytosis inhibitor pretreatments (the characteristics of applied primer sequences are detailed in **Supplementary Table 1**).

**Figure 5:**
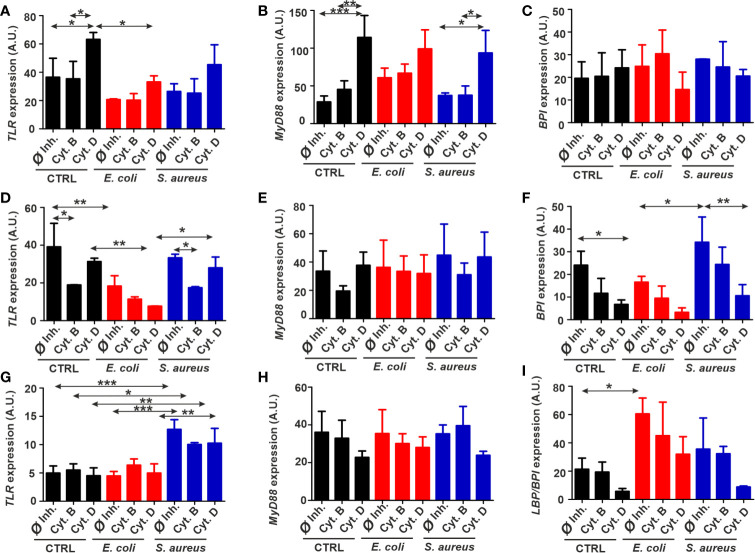
Fluctuations of immune-related gene expressions in target cells exposed to bacteria and phagocytosis inhibitors. Normalized expression of *TLR*, *MyD88*, and *BPI* in **(A–C)** THP-1, **(D–F)** diff. THP-1 and normalized expression of *TLR*, *MyD88*, and *LBP*/*BPI* **(G–I)** in coelomocytes after bacterial challenge in the absence or presence of the uptake inhibitors. The results are presented as mean ± SD of three replicates. Asterisks display statistical significance (*p < 0.05, **p < 0.01, ***p < 0.001). The significance of data was evaluated by one-way ANOVA with Tukey’s multiple comparison test. A.U., arbitrary units.

No significant elevations of *TLR* and *MyD88* levels by bacterial stimulation were revealed in THP-1 cells, although cytochalasin D increased their expression regardless of bacterial exposure (**Figures 5A, B**). *BPI* mRNA expression pattern was not affected by either condition (**Figure 5C**). Incubation with *E. coli* has downregulated *TLR* mRNA expression compared to the corresponding control samples in diff. THP-1 cells (**Figure 5D**). Cytochalasin B treatment evoked a slight decrease in *TLR* mRNA level (**Figure 5D**). In the case of *MyD88*, there were no significant changes (**Figure 5E**), while *BPI* mRNA level was elevated following *S. aureus* exposure and decreased upon actin disruption by cytochalasin D (**Figure 5F**).

In the case of coelomocytes, *TLR* mRNA expression was profoundly elevated only in the response towards *S. aureus*; however, neither of cytochalasins have changed this parameter (**Figure 5G**). No alteration in *MyD88* mRNA level was observed during any exposure conditions (**Figure 5H**). Nevertheless, *LBP/BPI* expression is significantly peaked in response to Gram-negative *E. coli* challenge and attenuated by cytochalasin D inhibition in every condition (**Figure 5I**).

## 4 Discussion

In vertebrates, myeloid and lymphoid cells are responsible for maintaining the immune response, while in invertebrates, immune functions are carried out by mesoderm-derived, specialized immune cells (e.g., coelomocytes, hemocytes). Earthworms, living in a microbe-rich environment, are constantly exposed to bacteria; their immune homeostasis is governed by several cellular and humoral components (23–25). Recent publications are more focused on the variations of immune-related genes upon microbial challenge in earthworms (26, 27). However, the different cellular engulfment routes to eliminate microbes by coelomocytes are rather unknown in earthworms, similarly to other invertebrate models.

In this study, we aimed to comparatively analyze the various endocytic pathways of bacterial internalization mediated by earthworm and human immune (THP-1) cells. THP-1 cell line proved to be a reliable model in our previous comparative *in vitro* study to observe nanoparticle toxicity against earthworm and human cells (28), and we favored this cell line over other monocyte/macrophage cell lines (J774, RAW264, etc.) to keep our comparative approach standard. Another benefit is that THP-1 monocytes can be differentiated into macrophage-like cells, which proved to resemble human macrophages at functional and phenotype levels (29). Hence, usage of the THP-1 cells line allowed us to investigate and compare bacterial uptake pathways in two human myeloid cell types. Another advantage of cell lines is their uniformity and simplicity in maintaining under standard laboratory conditions compared to primary cells. It is a valid concern that primary macrophages behave differently compared to a monocyte/macrophage cell line; however, a recent publication (30) has compared the bacterial uptake and inflammatory consequences (chemokine/cytokine mRNA expression and cytokine secretion) between human monocyte-derived macrophages and THP-1 cells and found no significant differences. In addition, another comparison (31) has revealed that THP-1 cell line can be applied as simple substitute for human macrophages in basic functional assays but should be avoided in fine-screening assays of certain drug candidates.

Earlier, we have applied (8, 26) heat-inactivated *E. coli* and *S. aureus* bacteria strains for *in vitro* phagocytosis/stimulation assays. We aimed to keep the reproducibility and comparability with these previous experiments applying the same bacterial strains. Indeed, earthworms tolerate well the microbe-rich environment. Now, there is an increasing body of information about earthworm bacterial community. Recent article by Pass et al. (32) has identified that *Proteobacteria*, *Actinobacteria*, *Bacteroidetes*, and *Acidobacteria* are dominant in the whole body of *Lumbricus rubellus* earthworms. These data was supported by the findings (33) from the gut of *Eisenia andrei*, with only the difference of the occurrence of *Firmicutes* phyla. Furthermore, *L. rubellus* microbiome is dominated by the genus *Serratia*, while in *E. andrei*, it is dominated by *Acinetobacter* genus. According to Dales and Kalaç (23), *Aeromonas hydrophila* and *Serratia marcescens* Gram-negative bacteria are potential natural pathogen bacteria in *E. fetida* earthworms; however, it is widely accepted that human pathogen bacteria are introduced into earthworms (or other invertebrates) (34, 35).

Initially, the kinetic analysis revealed that bacterial engulfment in THP-1, diff. THP-1 cells, and coelomocytes began at the first hour of incubation but to a different extent. According to the literature (29, 36), diff. THP-1 cells take up the bacteria to a greater extent than the undifferentiated THP-1 monocytes. Yet, in our results, the rates of uptake by THP-1 cells and diff. THP-1 cells were rather similar during kinetical analysis.

Compared to the human counterpart, we observed a slower rate of uptake by coelomocytes. This correlates with previous results (37), which also showed a maximum increase in antibacterial (lysozyme-like) activity in coelomic fluid upon 2 days of *in vivo* bacterial challenge. An earthworm-specific PRR, the coelomic cytolytic factor (CCF) that can recognize lipopolysaccharide (LPS), peptidoglycan, and β-1,3-glucan, is also evidenced to show an increased tendency after 24 h of bacterial challenge (6). Hence, this is a reliable representation of the humoral innate response of earthworms that is in line with our data focusing on bacterial uptake by coelomocytes.

We have monitored the passive transfer of bacteria into the cells, by incubation at 4°C, since at this temperature, the active transport, including phagocytosis, is blocked. The uptake was significantly reduced in the tested cell types (**Figures 1**–**3**), except during *S. aureus* uptake by coelomocytes (**Figure 3**). These results indicate that the internalization of *E. coli* and *S. aureus* is an energy-dependent process. Generally, earthworms flourish at 15–20°C, and their cells can tolerate better the lower temperature compared to mammalian cells. This may explain the maintained engulfment activity in coelomocytes at 4°C (38).

It is generally established that the particles larger than 500 nm, including most of the bacteria, are taken up by phagocytosis. In this regard, Veiga and Cossart (39) demonstrated that bacterial internalization might not be restricted only to phagocytic uptake, but other endocytosis pathways can be also encompassed. For example, some bacteria, including *E. coli*, manage to avoid degradation in lysosomes by entering the cell through caveolae-mediated endocytosis (40). Therefore, we started our experiments by testing different groups of inhibitors to target certain uptake pathways. By inhibiting the core components of the different endocytic pathways, it is possible to evaluate which of them participates in the endocytosis process. Albeit some of the pharmacological inhibitors have a weak specificity, they remain widely applied (14). They were chosen to target the major known cellular uptake pathways: macropinocytosis, clathrin- and caveolin-mediated endocytosis, and phagocytosis (1).

Amiloride and its derivative 5-(*N*-ethyl-*N*-isopropyl) amiloride (EIPA) was reported to inhibit macropinocytosis by blocking the Na^+^/H^+^ exchanger (14) and colchicine by disturbing microtubules in pinocytosis (41). In addition, since CvME involves cell membrane cholesterol, this endocytic pathway can be disrupted by cholesterol depletion (42). This is a mechanism of action for methyl-ß-cyclodextrin (MßCD), which possesses hydrophobicity resulting in a high affinity to membrane cholesterol and its depletion (43, 44). Antibiotic nystatin can induce changes in the shape of the caveolae and make aggregates of cholesterol leading to its sequestration (45). As for CME, amantadine was reported to interfere by blocking the budding of clathrin-coated pits (46). The aforementioned endocytosis inhibitors were ineffective for bacterial engulfment in the studied cell types; however, we observed a slight amantadine action on THP-1-mediated *S. aureus* internalization, which might imply that some endocytosis pathways are activated when the other is blocked.

Due to the core role of actin in phagosome formation, such F-actin depolymerizing agents, as cytochalasin B and D, can inhibit phagocytosis (47). Applying cytochalasin D, it turned out to be a universal inhibitor for the uptake of both bacterial strains in the studied cell types, suggesting that both *E. coli* and *S. aureus* are internalized *via* phagocytosis (**Figures 1**–**3**). Another inhibitor of phagocytosis, cytochalasin B also showed effectiveness in blocking the uptake of both bacterial types in coelomocytes (**Figure 3**). Unlike for earthworm immunocytes, in THP-1 and diff. THP-1 cells, cytochalasin B inhibited only the engulfment of *S. aureus* (**Figures 1**, **2**). These results correlate with previous data of *E. coli* engulfment inhibition by cytochalasin D in human monocyte-derived macrophages (48). Cytochalasin D also reduced the *Streptococcus pneumoniae* uptake by THP-1 cells (49). These results underline the crucial interactions of cytoskeleton rearrangement and phagocytosis in bacterial uptake by in fact different but evolutionarily and functionally similar earthworm and human immunocytes.

To this end, it is worth to point out the role of the actin filaments in various types of endocytosis. Our CLSM studies evidenced that fluorochrome-conjugated phalloidin binds to actin filaments of the cytoskeleton, resulting in the high imaging contrast of the filaments bundles, which was especially valuable in depicting the cytoskeleton changes after application of cytochalasins. As expected, cytochalasin D caused actin depolymerization, which disturbed actin-dependent phagocytic uptake of bacteria, keeping them out of the cells or on the surface of the cells (**Figures 1E**, **3E**). It is well known for mammalian cells that membrane extensions created by actin polymerization are necessary for phagocytosis and macropinocytosis, while the other uptake processes are considered not to involve actin cytoskeleton rearrangements (39, 50, 51). Here, our data underscore the role of actin-dependent phagocytosis of bacteria not only in human but also in invertebrate immunocytes. On the other hand, in this set of experiments, we have only concentrated on the inhibitors of actin polymerization, but phagocytosis is a complex process mediated by several other target molecules. For instance, it is known that inhibition of phosphoinositide 3-kinase (PI 3-kinase), protein kinase C, and Rho family of GTPases also results in reduced phagocytosis by vertebrate macrophages (52). In this regard, such inhibitors (e.g., wortmannin, LY290042, genistein, bisindolymaleimide, ML141) should be tested during future engulfment studies in earthworm coelomocytes, since limited data are available about those from invertebrate models (53, 54).

Applying LysoTracker dye to selectively stain the acidic organelles and the quantitative colocalization approach enables us to more precisely monitor bacterial fate in the cells. Microbial infections can increase the lysosome numbers in earthworm coelomocytes and elevate the activity of phagolysosomal markers (acid and alkaline phosphatase) in amoebocytes (8, 10). These data correlate with our recent CLSM images, where we observed colocalization of engulfed bacteria with lysosomes, suggesting acidification of these organelles and production of phagosomal enzymes. Manders’ coefficient values corresponding to bacteria/lysosome colocalization greatly decreased for cytochalasin D treated cells of all types, suggesting that lysosomes assist *E. coli* and *S. aureus* phagocytosis in both invertebrate and vertebrate immunocytes (**Figure 4**).

Innate immunity operates with a set of highly conserved pattern recognition receptors (PRRs). For instance, TLRs were first discovered in *Drosophila melanogaster* as a molecule important in the response to fungal and Gram-positive bacterial infections (55). TLR signaling in mammals shares similarities with *Drosophila*’s TLR-induced mechanisms. It is worth highlighting the role of the most conserved region, Toll/IL-1R (TIR) domain, a cytoplasmic part of TLR, which mediates the recruitment of adaptor proteins and starts the intracellular signaling cascade then leads to inflammatory cytokine production (56).

We have very limited information about the function of PRRs in earthworms (22), and we have relatively restricted research tools to test their involvement during earthworm immune response (7). Downstream inflammatory events occur following pathogen ligand and PRR engagements (27); however, cytokine homologs have not been yet identified in earthworms. Potential inflammatory candidates would be lysenin (8) and the pattern recognition receptor CCF (57); however, those molecules have no known human (vertebrate) homologs. To keep the comparative approach, we decided to choose only those immune-related genes (*TLR*, *MyD88*, and *LBP/BPI*) that we can test in both models.

TLR was recently identified in *E. andrei* earthworms (*Ea*TLR), and it is modulated upon bacteria stimulation (58). Hence, since it resembles structural and functional similarity to its human counterpart, we aimed to identify *TLR* expression upon the application of cytochalasin B and D and incubation with bacteria.

Bacterial stimulation did not elevate the *TLR* mRNA expression levels in THP-1, and in the case of diff. THP-1, *TLR* was even downregulated following the incubation with *E. coli*. Since cytochalasin B was reported to show anti-inflammatory responses, its impact is noticeable in decreased immune-related genes’ expression (11), which coincides with *TLR* mRNA downregulation in diff. THP-1 cells of our experiments (**Figure 5D**). In contrast, while cytochalasin D treatment could stop bacteria from entering, it did not prevent the TLR signaling. Since cytochalasin D is also able to upregulate nuclear factor kappa B (NF-κB) levels (59), this might correlate with TLR expression. Nevertheless, it just confirms that the relationships between TLR levels, actin cytoskeleton, and intracellular signaling are still poorly understood.

*TLR* expression in *S. aureus*-exposed coelomocytes showed a significant increase (**Figure 5G**), which supports earlier results (58) where a fourfold change in *EaTLR* expression was reported after *Bacillus subtilis* stimulation. In contrast, incubation with *E. coli* did not significantly amplify the *EaTLR* expression that is in line with our observations. Since cytochalasins could not markedly reduce *EaTLR* levels, we affirm that actin depolymerization hinders bacterial engulfment in coelomocytes but does not change the capacity of TLR for the engagements with bacterial ligands.

After ligand binding of TLR, MyD88 adaptor triggers downstream signaling cascades, leading to NF-κB and mitogen-activated protein kinase (MAPK) activation and inflammatory cytokine production (60). However, a MyD88-independent pathway was reported by Kawai et al. (61), where they demonstrated that despite impaired cytokine production, MyD88-deficient macrophages had normal NF-κB signaling in response to LPS. In our experiments, bacterial stimulation did not provoke the elevation of *MyD88* expression compared to Ø inhibitor controls (**Figure 5**), which might suggest the involvement of MyD88-independent mechanism in these phagocytic processes.

Furthermore, there is a connection between activation of one or another pathway and the state of the actin cytoskeleton in human macrophages. TLR induces phagocytic gene expression through the MyD88 pathway without the involvement of actin. In turn, a second mechanism is MyD88-independent but requires actin for activation of this pathway (62). This provides the evidence of MyD88-independent pathway involvement in the process of bacterial uptake. Upon the impairment of actin polymerization by cytochalasins, an alternative activation of actin-independent MyD88 is probable. Besides, we cannot rule out that *MyD88* expression variations occurred at the earlier time points. In this case, our results might be connected to negative feedback regulatory processes to control excessive TLR-mediated mechanisms in the later stages of the infections (63–65).

Even though the MyD88-dependent/independent TLR pathway is well conserved across the evolution (66), limited data for *E. andrei* are available regarding the MyD88 involvement in earthworm immune response. Whole transcriptome analysis of *E. andrei* has identified several immune-related sequences, including MyD88 as an adaptive molecule for MAPK signaling activated by TLR (67). As our results suggest, there is no significant difference between *MyD88* expression levels in unstimulated and bacteria stimulated coelomocytes. Yet, in oppose to THP-1 and diff. THP-1 cells data, cytochalasins show a tendency to downregulate *MyD88* mRNA expression in coelomocytes, which is in line with decreased phagocytosis of bacteria (**Figure 5H**). This could propose that just like in *D. melanogaster*, MyD88 in coelomocytes is located in the plasma membrane (68) and can be disrupted by cytochalasins. Therefore, in earthworms, actin cytoskeleton rearrangement may be a crucial event in the regulation of innate immune responses that control TLRs and their downstream signaling proteins.

The BPI, which, in human macrophages, is known to possess antimicrobial functions, is known to be less expressed in human monocytes (69) that was also observed in our experiments. Unexpectedly, diff. THP-1 cells stronger reacted to Gram-positive than to Gram-negative bacteria (**Figure 5F**). Nevertheless, cytochalasin D affected *BPI* mRNA levels, indicating that BPI is located in the plasma membrane and might also directly participate in the bacterial uptake.

The homolog of this well-conserved PRR was also identified in *E. andrei* (70). Later, our research group showed that *EaLBP/BPI* mRNA is specifically expressed in the amoebocyte subset (71). Previous data have revealed upregulation of *EaLBP/BPI* gene transcription after stimulation with bacteria, including *E. coli* (70), which is in line with our findings. For *S. aureus*, there might be another PRR involved in its recognition and signal transduction, for example, CCF known to be more specific for Gram-positive bacteria (57).

To conclude, we applied *E. coli* and *S. aureus* bacteria to characterize the microbial uptake routes in invertebrate and vertebrate immunocytes. Despite some differences in the intracellular signaling, the conserved actin-dependent phagocytosis suggests the functional similarities of bacterial internalization process for earthworm and human immunocytes.

## Data Availability Statement

The original contributions presented in the study are included in the article/**Supplementary Material**. Further inquiries can be directed to the corresponding author.

## Author Contributions

Conceptualization: BK and PE. Methodology: BK, KB, GS, and PE. Investigation: BK, KB, and GS. Writing—original draft preparation: BK. Writing—review and editing: KB, GS, PN, and PE. Supervision: PE. Funding acquisition: PE and KB. All authors contributed to the article and approved the submitted version.

## Funding

This research was funded by the Medical School Research Foundation University of Pécs (PTE-ÁOK-KA 2017/4), GINOP-232-15-2016-00050 and EFOP-361-16-2016-00004. The purchase of the Olympus FV-1000 laser scanning confocal system was supported by grant GVOP-3.2.1-2004-04-0172/3.0 to the University of Pécs. TKP2020-IKA-08 has been implemented with the support provided from the National Research, Development and Innovation Fund of Hungary, financed under the 2020-4.1.1-TKP2020 funding scheme. The work was supported by the ÚNKP-19-3-I New National Excellence Program of the Ministry for Innovation and Technology and by the György Romhányi Research Scholarship of the Medical School, University of Pécs.

## Conflict of Interest

The authors declare that the research was conducted in the absence of any commercial or financial relationships that could be construed as a potential conflict of interest.

## Publisher’s Note

All claims expressed in this article are solely those of the authors and do not necessarily represent those of their affiliated organizations, or those of the publisher, the editors and the reviewers. Any product that may be evaluated in this article, or claim that may be made by its manufacturer, is not guaranteed or endorsed by the publisher.

## References

[1] Conner SD Schmid SL. Regulated Portals of Entry Into the Cell. Nature (2003) 422:37–44. 10.1038/nature01451 12621426

[2] Hall A Nobes CD. Rho GTPases: Molecular Switches That Control the Organization and Dynamics of the Actin Cytoskeleton. Philos Trans R Soc Lond B Biol Sci (2000) 355:965–70. 10.1098/rstb.2000.0632 PMC1692798 11128990

[3] DeDuve C. Blueprint for a Cell: The Nature and Origin of Life. 1st edition. Burlington, N.C: Carolina Biological Supply Co (1991).

[4] Hartenstein V Martinez P. Phagocytosis in Cellular Defense and Nutrition: A Food-Centered Approach to the Evolution of Macrophages. Cell Tissue Res (2019) 377:527–47. 10.1007/s00441-019-03096-6 PMC6750737 31485720

[5] Arroyo Portilla C Tomas J Gorvel JP Leoulard H. From Species to Regional and Local Specialization of Intestinal Macrophages. Front Cell Dev Biol (2021) 8:624213. 10.3389/fcell.2020.624213 33681185 PMC7930007

[6] Bilej M Baetselier PD Dijck EV Stijlemans B Colige A Beschin A. Distinct Carbohydrate Recognition Domains of an Invertebrate Defense Molecule Recognize Gram-Negative and Gram-Positive Bacteria. J Biol Chem (2001) 276:45840–7. 10.1074/jbc.M107220200 11585829

[7] Engelmann P Bodó K Najbauer J Németh P. Annelida: Oligochaetes (Segmented Worms): Earthworm Immunity, Quo Vadis? Advances and New Paradigms in the Omics Era. In: EL Cooper, editor. Advances in Comparative Immunology. Cham: Springer International Publishing (2018). p. 135–59. 10.1007/978-3-319-76768-0_6

[8] Engelmann P Hayashi Y Bodó K Ernszt D Somogyi I Steib A. Phenotypic and Functional Characterization of Earthworm Coelomocyte Subsets: Linking Light Scatter-Based Cell Typing and Imaging of the Sorted Populations. Dev Comp Immunol (2016) 65:41–52. 10.1016/j.dci.2016.06.017 27349970

[9] Park EK Jung HS Yang HI Yoo MC Kim C Kim KS. Optimized THP-1 Differentiation Is Required for the Detection of Responses to Weak Stimuli. Inflammation Res (2007) 56:45–50. 10.1007/s00011-007-6115-5 17334670

[10] Engelmann P Molnár L Pálinkás L Cooper EL Németh P. Earthworm Leukocyte Populations Specifically Harbor Lysosomal Enzymes That may Respond to Bacterial Challenge. Cell Tissue Res (2004) 316:391–401. 10.1007/s00441-004-0874-x 15138884

[11] Kim MY Kim JH Cho JY. Cytochalasin B Modulates Macrophage-Mediated Inflammatory Responses. Biomol Ther (Seoul) (2014) 22:295–300. 10.4062/biomolther.2014.055 25143807 PMC4131529

[12] DeFife KM Jenney CR Colton E Anderson JM. Cytoskeletal and Adhesive Structural Polarizations Accompany IL-13-Induced Human Macrophage Fusion. J Histochem Cytochem (1999) 47:65–74. 10.1177/002215549904700107 9857213

[13] Valberg PA Brain JD Kane D. Effects of Colchicine or Cytochalasin B on Pulmonary Macrophage Endocytosis. Vivo J Appl Physiol (1981) 50:621–9. 10.1152/jappl.1981.50.3.621 7195895

[14] Ivanov AI. “Pharmacological Inhibition of Endocytic Pathways: Is It Specific Enough to be Useful”? In: Ivanov AI, editor. Exocytosis and Endocytosis. Methods in Molecular Biology, *vol.* 440. New York, USA: Humana Press (2008). p. 15–33. 10.1007/978-1-59745-178-9_2 18369934

[15] Van Hamme E Dewerchin HL Cornelissen E Verhasselt B Nauwynck HJ. Clathrin- and Caveolae-Independent Entry of Feline Infectious Peritonitis Virus in Monocytes Depends on Dynamin. J Gen Virol (2008) 89:2147– 56. 10.1099/vir.0.2008/001602-0 18753224

[16] Atger VM de la Llera Moya M Stoudt GW Rodrigueza WV Phillips MC Rothblat GH. Cyclodextrins as Catalysts for the Removal of Cholesterol From Macrophage Foam Cells. J Clin Invest (1997) 99:773–80. 10.1172/JCI119223 PMC507862 9045882

[17] Florian P Macovei A Sima L Nichita N Mattsby-Baltzer I Roseanu A. Endocytosis and Trafficking of Human Lactoferrin in Macrophage-Like Human THP-1 Cells. Biochem Cell Biol (2012) 90:449–55. 10.1139/o11-090 22380846

[18] Gliga AR Skoglund S Wallinder IO Fadeel B Karlsson HL. Size-Dependent Cytotoxicity of Silver Nanoparticles in Human Lung Cells: The Role of Cellular Uptake, Agglomeration and Ag Release. Part Fibre Toxicol (2014) 11:11. 10.1186/1743-8977-11-11 24529161 PMC3933429

[19] Bolte S Cordelières FP. A Guided Tour Into Subcellular Colocalization Analysis in Light Microscopy. J Microsc (2006) 224:213–32. 10.1111/j.1365-2818.2006.01706.x 17210054

[20] Manders EMM Verbeek FJ Aten JA. Measurement of Co Localization of Objects in Dual Colour Confocal Images. J Microsc (1993) 169:375–82. 10.1111/j.1365-2818.1993.tb03313.x 33930978

[21] Rorat A Kachamakova-Trojanowska N Jozkowicz A Kruk J Cocquerelle C Vandenbulcke F. Coelomocyte-Derived Fluorescence and DNA Markers of Composting Earthworm Species. J Exp Zool (2014) 321A:28–40. 10.1002/jez.1834 24115405

[22] Prochazková P Roubalova R Dvorák J Navarro Pacheco NI Bilej M. Pattern Recognition Receptors in Annelids. Dev Comp Immunol (2020) 102:103493. 10.1016/j.dci.2019.103493 31499098

[23] Dales RP Kalaç Y. Phagocytic Defence by the Earthworm *Eisenia foetida* Against Certain Pathogenic Bacteria. Comp Biochem Physiol (1992) 101A:487–90. 10.1016/0300-9629(92)90499-G

[24] Cooper EL Kauschke E Cossarizza A. Digging for Innate Immunity Since Darwin and Metchnikoff. BioEssays (2002) 24:319–33. 10.1002/bies.10077 11948618

[25] Engelmann P Cooper EL Németh P. Anticipating Innate Immunity Without a Toll. Mol Immunol (2005) 42:931–42. 10.1016/j.molimm.2004.09.038 15829285

[26] Bodó K Boros Á Rumpler É Molnár L Böröcz K Németh P. Identification of Novel Lumbricin Homologues in *Eisenia andrei* Earthworms. Dev Comp Immunol (2019) 90:41–6. 10.1016/j.dci.2018.09.001 30179632

[27] Dvořák J Roubalová R Procházková P Rossmann P Škanta F Bilej M. Sensing Microorganisms in the Gut Triggers the Immune Response in *Eisenia andrei* Earthworms. Dev Comp Immunol (2016) 57:67–74. 10.1016/j.dci.2015.12.001 26684064

[28] Hayashi Y Engelmann P Foldbjerg R Szabó M Somogyi I Pollák E. Earthworms and Humans *In Vitro*: Characterizing Evolutionarily Conserved Stress and Immune Responses to Silver Nanoparticles. Environ Sci Technol (2012) 46:4166–73. 10.1021/es3000905 22432789

[29] Daigneault M Preston JA Marriott HM Whyte MKB Dockrell DH. The Identification of Markers of Macrophage Differentiation in PMA-Stimulated THP-1 Cells and Monocyte-Derived Macrophages. PLoS One (2010) 5:e8668. 10.1371/journal.pone.0008668 20084270 PMC2800192

[30] Madhvi A Mishra H Leisching GR Mahlobo PZ Baker B. Comparison of Human Monocyte Derived Macrophages and THP-1 Like Macrophages as *In Vitro* Models for *M. tuberculosis* Infection. Comp Immunol Microbiol Infect (2019) 67:101355. 10.1016/j.cimid.2019.101355 31586851

[31] Tedesco S De Majo F Kim J Trenti A Trevisi L Fadini GP. Convenience Versus Biological Significance: Are PMA-Differentiated THP-1 Cells Are Reliable Substitute for Blood-Derived Macrophages When Studying *In Vitro* Polarization? Front Pharmacol (2018) 9:71. 10.3389/fphar.2018.00071 29520230 PMC5826964

[32] Pass DA Morgan AJ Read DS Filed D Weightman AJ Kille P. The Effect of Anthropogenic Arsenic Contamination on the Earthworm Microbiome. Environ Microbiol (2015) 17:1884–96. 10.1111/1462-2920.12712 25404571

[33] Aira M Bybee S Pérez-Losada M Dominguez J. Feeding on Microbiomes: Effects of Detritivory on the Taxonomic and Phylogenetic Bacterial Composition of Animal Manures. FEMS Microbiol Ecol (2015) 91:fiv117. 10.1093/femsec/fiv117 26432803

[34] Roubalová R Procházková P Hanč A Dvořák J Bilej M. Mutual Interactions of *E. andrei* Earthworms and Pathogens During the Process of Vermicomposting. Envrion Sci Pollut Res Int (2020) 27:33429–37. 10.1007/s11356-019-04329-5 30840250

[35] Kaito C Murakami K Imai L Furuta K. Animal Infection Models Using non-Mammals. Microbiol Immunol (2020) 64:585–92. 10.1111/1348-0421.12834 PMC7590188 32757288

[36] Schwende H Fitzke E Ambs P Dieter P. Differences in the State of Differentiation of THP-1 Cells Induced by Phorbol Ester and 1,25-Dihydroxyvitamin D3. J Leuk Biol (1996) 59:555–61. 10.1002/jlb.59.4.555 8613704

[37] Köhlerová P Beschin A Šilerová M De Baetselier P Bilej M. Effect of Experimental Microbial Challenge on the Expression of Defense Molecules in *Eisenia foetida* Earthworm. Dev Comp Immunol (2004) 28:701–11. 10.1016/j.dci.2004.01.001 15043940

[38] Molnár L Engelmann P Somogyi I Mácsik LL Pollák E. Cold-Stress Induced Formation of Calcium and Phophorous Rich Chloragocyte Granules (Chloragosomes) in the Earthworm *Eisenia foetida* . Comp Biochem Physiol (2012) 163A:199–209. 10.1016/j.cbpa.2012.06.005 22710253

[39] Veiga E Cossart P. The Role of Clathrin-Dependent Endocytosis in Bacterial Internalization. Trends Cell Biol (2006) 16:499–504. 10.1016/j.tcb.2006.08.005 16962776 PMC7126422

[40] Shin JS Abraham SN. Caveolae as Portals of Entry for Microbes. Microb Infect (2001) 3:755–61. 10.1016/S1286-4579(01)01423-X 11489424

[41] Starling D Duncan R Lloyd JB. The Role of Microtubules in Pinocytosis. Inhibition of Fluid-Phase Pinocytosis in the Rat Visceral Yolk Sac by Mitoclasic and Related Agents. Cell Biol Int Rep (1983) 7:593–602. 10.1016/0309-1651(83)90113-3 6616627

[42] Razani B Woodman SE Lisanti MP. Caveolae: From Cell Biology to Animal Physiology. Pharmacol Rev (2002) 54:431–67. 10.1124/pr.54.3.431 12223531

[43] Irie T Fukunaga K Pitha J. Hydroxypropylcyclodextrins in Parenteral Use. I: Lipid Dissolution and Effects on Lipid Transfers *In Vitro* . J Pharm Sci (1992) 81:521–3. 10.1002/jps.2600810609 1522487

[44] Kilsdonk EP Yancey PG Stoudt GW Bangerter FW Johnson WJ Phillips MC. Cellular Cholesterol Efflux Mediated by Cyclodextrins. J Biol Chem (1995) 270:17250–6. 10.1074/jbc.270.29.17250 7615524

[45] Ros-Baro A Lopez-Iglesias C Peiro S Bellido D Palacin M Zorzano A. Lipid Rafts Are Required for GLUT4 Internalization in Adipose Cells. Proc Natl Acad Sci USA (2001) 98:12050–5. 10.1073/pnas.211341698 PMC59765 11593015

[46] Perry DG Daugherty GL Martin WJ. Clathrin-Coated Pit-Associated Proteins Are Required for Alveolar Macrophage Phagocytosis. J Immunol (1999) 162:380–6. 9886410

[47] Peterson JR Mitchison TJ. Small Molecules, Big Impact: A History of Chemical Inhibitors and the Cytoskeleton. Chem Biol (2002) 9:1275–85. 10.1016/s1074-5521(02)00284-3 12498880

[48] Peiser L Gough PJ Kodama T Gordon S. Macrophage Class A Scavenger Receptor-Mediated Phagocytosis of *Escherichia coli*: Role of Cell Heterogeneity, Microbial Strain, and Culture Conditions *In Vitro* . Infect Immun (2000) 68:1953–63. 10.1128/iai.68.4.1953-1963.2000 PMC97372 10722588

[49] Kohler TP Scholz A Kiachludis D Hammerschmidt S. Induction of Central Host Signaling Kinases During Pneumococcal Infection of Human THP-1 Cells. Front Cell Infect Microbiol (2016) 6:48. 10.3389/fcimb.2016.00048 27200303 PMC4844997

[50] Ayscough KR. Endocytosis and the Development of Cell Polarity in Yeast Require a Dynamic F-Actin Cytoskeleton. Curr Biol (2000) 10:1587–90. 10.1016/s0960-9822(00)00859-9 11137010

[51] Fujimoto LM Roth R Heuser JE Schmid SL. Actin Assembly Plays a Variable, But Not Obligatory Role in Receptor-Mediated Endocytosis in Mammalian Cells. Traffic (2000) 1:161–71. 10.1034/j.1600-0854.2000.010208.x 11208096

[52] Aderem A Underhill DM. Mechanisms of Phagocytosis in Macrophages. Ann Rev Immunol (1999) 17:593–623. 10.1146/annurev.immunol.17.1.593 10358769

[53] Plows LD Cook RT Davies AJ Walker AJ. Phagocytosis of *Lymnae stagnalis* Haemocytes: A Potential Role for Phosphatidylinositol 3-Kinase But Not Protein Kinase A. J Inverteb Pathol (2006) 91:74–7. 10.1016/j.jip.2005.10.011 16376929

[54] De Winter P Rayne RC Coast GM. The Effects of Intracellular Signaling Pathway Inhibitors on Phagocytosis by Haemocytes of *Manduca sexta* . J Insect Physiol (2007) 53:975–82. 10.1016/j.jinsphys.2007.04.001 17597143

[55] Michel T Reichhart JM Hoffmann JA Royet J. *Drosophila* Toll Is Activated by Gram-Positive Bacteria Through a Circulating Peptidoglycan Recognition Protein. Nature (2001) 414:756–9. 10.1038/414756a 11742401

[56] Takeda K Akira S. Toll Receptors and Pathogen Resistance. Cell Microbiol (2003) 5:143–53. 10.1046/j.1462-5822.2003.00264.x 12614458

[57] Beschin A Bilej M Hanssens F Raymakers J Van Dyck E Revets H. Identification and Cloning of a Glucan- and Ipopolysaccharide-Binding Protein From *Eisenia foetida* Earthworm Involved in the Activation of Prophenoloxidase Cascade. J Biol Chem (1998) 273:24948–54. 10.1074/jbc.273.38.24948 9733802

[58] Škanta F Roubalová R Dvořák J Procházková P Bilej M. Molecular Cloning and Expression of TLR in the *Eisenia andrei* Earthworm. Dev Comp Immunol (2013) 41:694–702. 10.1016/j.dci.2013.08.009 23969138

[59] Rosette C Karin M. Cytoskeletal Control of Gene Expression: Depolymerization of Microtubules Activates NF-Kappa B. J Cell Biol (1995) 128:1111–9. 10.1083/jcb.128.6.1111 PMC2120413 7896875

[60] Akira S Takeda K Kaisho T. Toll-Like Receptors: Critical Proteins Linking Innate and Acquired Immunity. Nat Immunol (2001) 2:675–80. 10.1038/90609 11477402

[61] Kawai T Adachi O Ogawa T Takeda K Akira S. Unresponsiveness of MyD88-Deficient Mice to Endotoxin. Immunity (1999) 11:115–22. 10.1016/S1074-7613(00)80086-2 10435584

[62] Kong L Ge BX. MyD88-Independent Activation of a Novel Actin-Cdc42/Rac Pathway Is Required for Toll-Like Receptor-Stimulated Phagocytosis. Cell Res (2008) 18:745–55. 10.1038/cr.2008.65 18542102

[63] Brint EK Xu D Liu H Dunne A McKenzie ANJ O’Neill LAJ. ST2 Is an Inhibitor of Interleukin 1 Receptor and Toll-Like Receptor 4 Signaling and Maintains Endotoxin Tolerance. Nat Immunol (2004) 5:373–9. 10.1038/ni1050 15004556

[64] Janssens S Burns K Tschopp J Beyaert R. Regulation of Interleukin-1- and Lipopolysaccharide-Induced NF-KappaB Activation by Alternative Splicing of Myd88. Curr Biol (2002) 12:467–71. 10.1016/s0960-9822(02)00712-1 11909531

[65] Liew FY Xu D Brint EK O’Neill LAJ. Negative Regulation of Toll-Like Receptor-Mediated Immune Responses. Nat Rev Immunol (2005) 5:446–58. 10.1038/nri1630 15928677

[66] Yang M Yuan S Huang S Li J Xu L Huang H. Characterization of bbtTICAM From Amphioxus Suggests the Emergence of a MyD88-Independent Pathway in Basal Chordates. Cell Res (2011) 21:1410–23. 10.1038/cr.2011.156 PMC3193451 21931360

[67] Mikami Y Fukushima A Kuwada-Kusunose T Sakurai T Kitano T Komiyama Y. Whole Transcriptome Analysis Using Next-Generation Sequencing of Sterile-Cultured *Eisenia andrei* for Immune System Research. PLoS One (2015) 10:e0118587. 10.1371/journal.pone.0118587 25706644 PMC4338202

[68] Marek LR Kagan JC. Phosphoinositide Binding by the Toll Adaptor Dmyd88 Controls Antibacterial Responses in Drosophila. Immunity (2012) 36:612–22. 10.1016/j.immuni.2012.01.019 PMC3354765 22464168

[69] Balakrishnan A Schnare M Chakravortty D. Of Men Not Mice: Bactericidal/Permeability-Increasing Protein Expressed in Human Macrophages Acts as a Phagocytic Receptor and Modulates Entry and Replication of Gram-Negative Bacteria. Front Immunol (2016) 7:455. 10.3389/fimmu.2016.00455 27822215 PMC5075746

[70] Škanta F Procházková P Roubalová R Dvořák J Bilej M. LBP/BPI Homologue in *Eisenia andrei* Earthworms. Dev Comp Immunol (2016) 54:1–6. 10.1016/j.dci.2015.08.008 26297397

[71] Bodó K Ernszt D Németh P Engelmann P. Distinct Immune-and Defense-Related Molecular Fingerprints in Sepatated Coelomocyte Subsets of *Eisenia andrei* Earthworms. Inv Surv J (2018) 15:338–45. 10.25431/1824-307X/isj.v15i1.338-345

