# Supplementary material for: Bacterial Engulfment Mechanism Is Strongly Conserved in Evolution Between Earthworm and Human Immune Cells
Source: Front Immunol. 2021 Sep 1;12:733541. doi: 10.3389/fimmu.2021.733541 (PMC8440998; doi:10.3389/fimmu.2021.733541)
Supplement: Supplementary file 1 [file DataSheet_1.pdf]

frontiers

Supplementary Material

TABLE OF CONTENTS

1. Materials and methods **2**
1.1. RNA-isolation, cDNA synthesis and real-time PCR **2**
2. References **3**
3. Supplementary tables **4**
4. Supplementary figures **5**

1. Materials and methods

1.1. RNA-isolation, cDNA synthesis and real-time PCR

Total RNA was extracted from the THP-1, diff. THP-1 cells and coelomocytes applying a NucleoSpin RNA Mini kit (Macherey-Nagel GmbH, Düren, Germany) according to the manufacturer's instructions. Following the elution of RNA in nuclease-free water, its quantity and quality were measured with a NanoDrop spectrophotometer at 260 nm. RNA samples were stored at -80°C. Prior to cDNA synthesis, DNase I digestion (Amplification Grade DNase I; Sigma-Aldrich) was executed (25°C for 15 min, 72°C for 10 min). A High Capacity cDNA Reverse Transcription Kit (Thermo Scientific) was applied for cDNA synthesis according to the manufacturer's protocol. Prepared cDNAs were stored at -20°C and subsequently used as qPCR reaction templates.

Gene-specific primers (please see Supplementary Table 1) were designed by Primer Express Software (Thermo Scientific) as we described earlier (1, 2). Target mRNA expressions were measured by qPCR using a Maxima SYBR Green Master Mix (Thermo Scientific) with an ABI Prism 7500 (Applied Biosystems). Thermal profile started at 95°C and lasted for 10 minutes. Subsequently, it was followed by 40 cycles of denaturation (35 s at 95°C), hybridization (35 s at 58°C), and elongation (1 min at 72°C) stages with ultimately a dissociation step. Each reaction was performed in a volume of 25 µL and samples were tested in triplicates. Quantitative measurements were normalized to *TATA binding-protein* (*TBP*) or *RPL17* mRNA level (1, 2). PCR data were determined from three independent experiments.

2. References

1. Szabo M, Sárosi V, Balikó Z, Bodó K, Farkas N, Berki T, Engelmann P. Deficiency of innate-like T lymphocytes in chronic obstructive pulmonary disease. *Resp Res* (2017) 28: 197. https://doi.org/10.1186/s12931-017-0671-1

2. Bodó K, Ernszt D, Németh P, Engelmann P. Distinct immune- and defense-related molecular fingerprints in separated coelomocyte subsets in *Eisenia andrei* earthworms. *Invertebr Surv J* (2018) 15: 338-345. https://doi.org/10.25431/1824-307X/isj.v15i1.338-345.

3. Supplementary tables

Table 1. Characteristics of earthworm and human primer sequences applied for qPCR analysis

Species	Target gene	Gene Bank Accession #	Sequence (5'-3')[a]	Amplicon size (bp)
Eisenia andrei	*RPL 17*	BB998250	GCA GAA TTC AAG GGA CTG GA<br>CTC CTT CTC GGA CAG GAT GA	159
	TLR	JX898685	ATT GTG TCA AAC GCC TTC GC<br>GTC GGC GAT CTC TTC CAA CA	123
	MyD88	EH670202	TGC GAG TAC AGG CTC GTT AAC<br>CGT GCA GAT GTG GTT TAG GA	100
	LBP/BPI	JQ407018	GGT TCG ACC TCC GAC GAT AC<br>GGT CAA CAG GGC GTC CAT TA	107
Homo sapiens	*TBP*	BC110341	CCA GAC TGG CAG CAA GAA AAT<br>TCA CAG CTC CCC ACC ATA TTC	100
	TLR	NM_003266	AAA GCC GAA AGG TGA TTG TTG T<br>ACT GCC AGG TCT GAG CAA TCT C	90
	MyD88	NM_0024688	TGA CTT CCA GAC CAA ATT TGC A<br>GAA CTC TTT CTT CAT TGC CTT GTA CT	94
	BPI	NM_001725	TGGCATGCACACAACTGGTT<br>AGTTCCAGGAGCAGCCTATCC	90

[a]Upper and lower primer sequences indicate forward and reverse primers

4. Supplementary figures

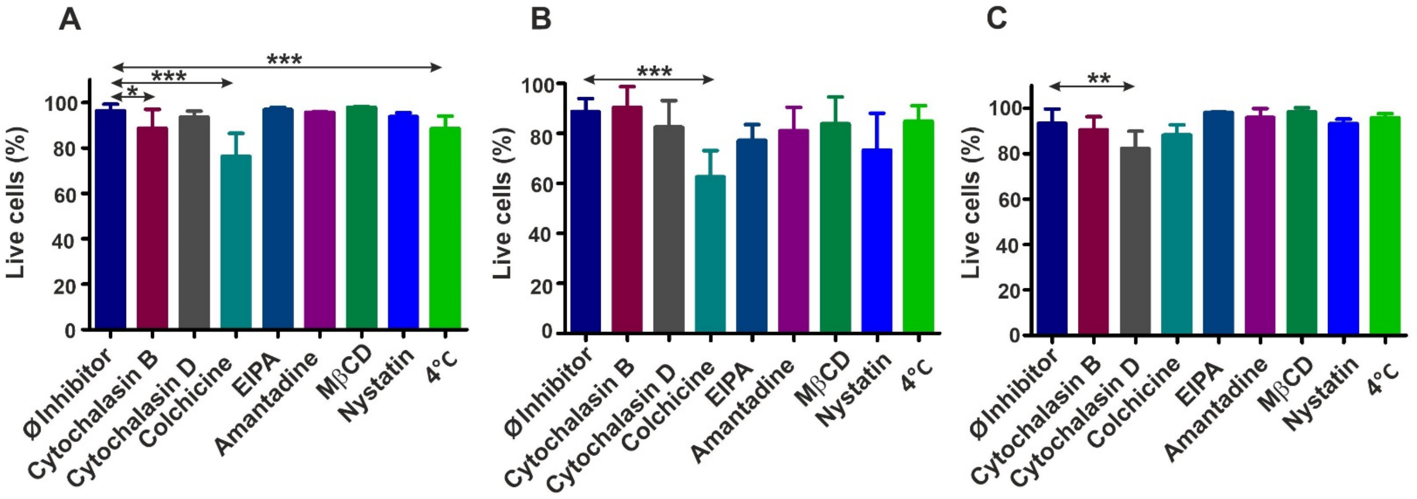

Supplementary Figure 1. Survival rate of THP-1 cells (**A**), diff. THP-1 cells (**B**) and coelomocytes (**C**) following uptake inhibition. Cytotoxicity of various treatments was observed by 7-AAD live/dead cell assay by flow cytometry following 24 h incubation. Results are presented as mean ± SD, n=4. Asterisks denote statistical significance (*p<0.05, **p<0.01, ***p<0.001) between the Ø inhibitor control and different treatments.

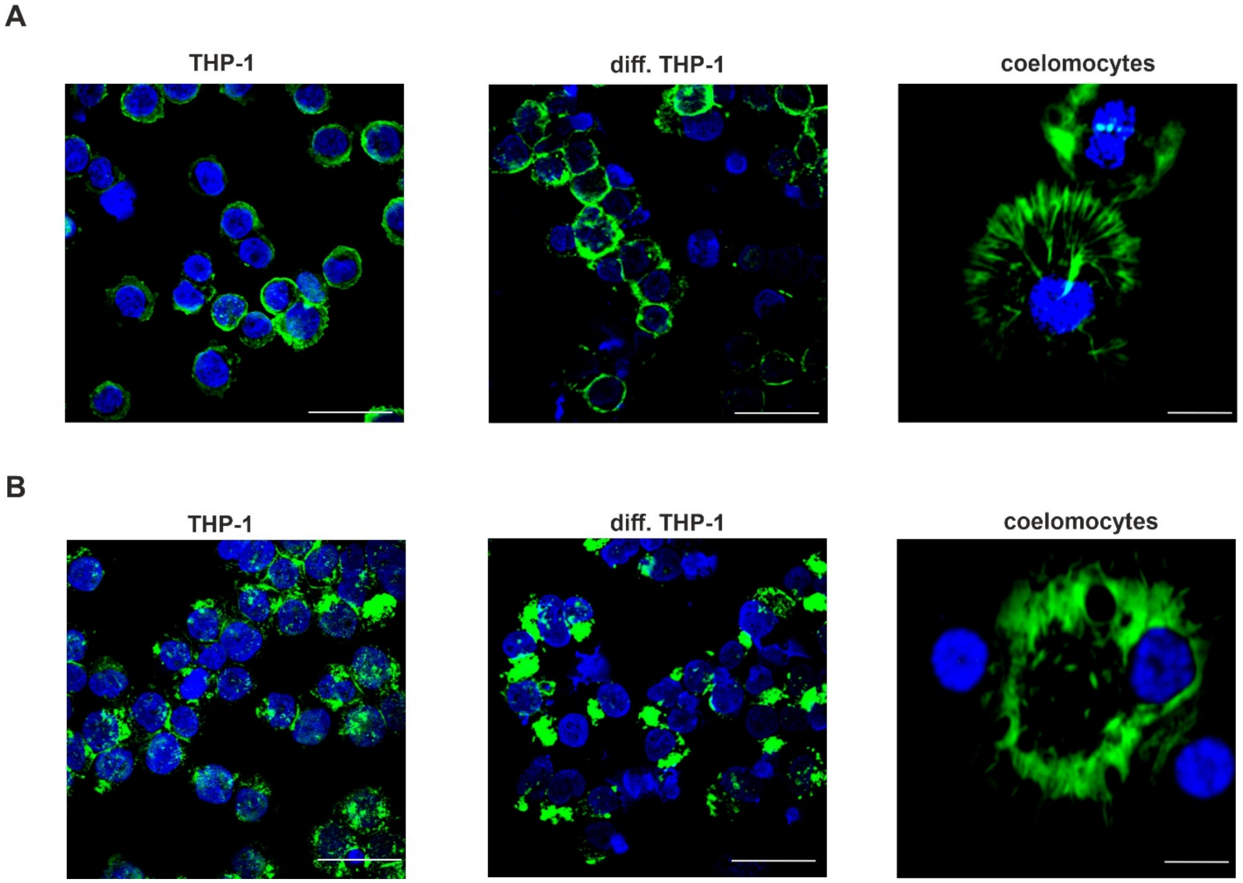

Supplementary Figure 2. Representative CLSM images of Ø inhibitor control (**A**) and 5 µM cytochalasin D exposed (**B**) THP-1 cells, diff. THP-1 cells and coelomocytes. Actin filaments were visualized with AlexaFluor 488 phalloidin (green). Note the biased polymerization of actin filaments following cytochalasin D treatments. Nuclear counterstaining was performed with DAPI (blue). Scale bars: 50 µm (THP-1, diff.THP-1), 10 µm (coelomocytes).

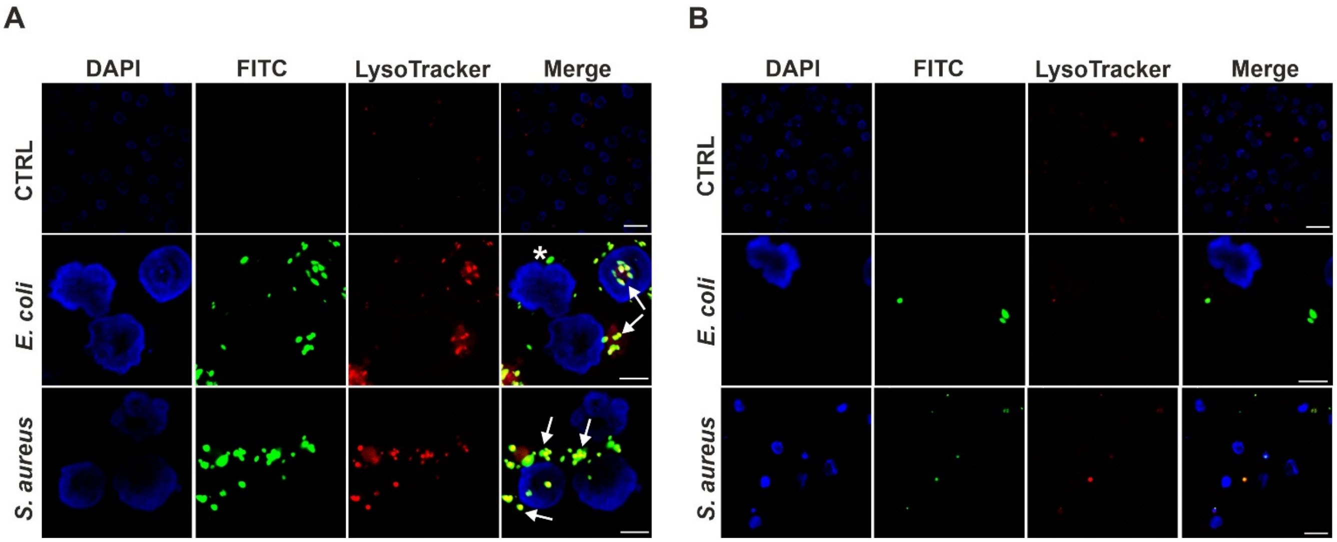

Supplementary Figure 3. Representative CLSM images of THP-1 cells exposed to FITC-conjugated *E. coli* or *S. aureus* bacteria (green) strains. Subsequently, LysoTracker staining was applied to visualize colocalized engulfed bacteria with lysosomes (red) in THP-1 cells. Clear colocalization can be observed in THP-1 cells without inhibitors (**A**, arrows), while in the presence of 5 µM cytochalasin D (**B**) this process is biased. Note the surface-bound bacteria (**A**, asterisk). Nuclear counterstaining was performed with DAPI (blue). Scale bars: 50 µm (CTRL - **A**, **B**; *S. aureus*-**B**), 10 µm (*E. coli*-**A**, **B**; *S. aureus* - **A**).

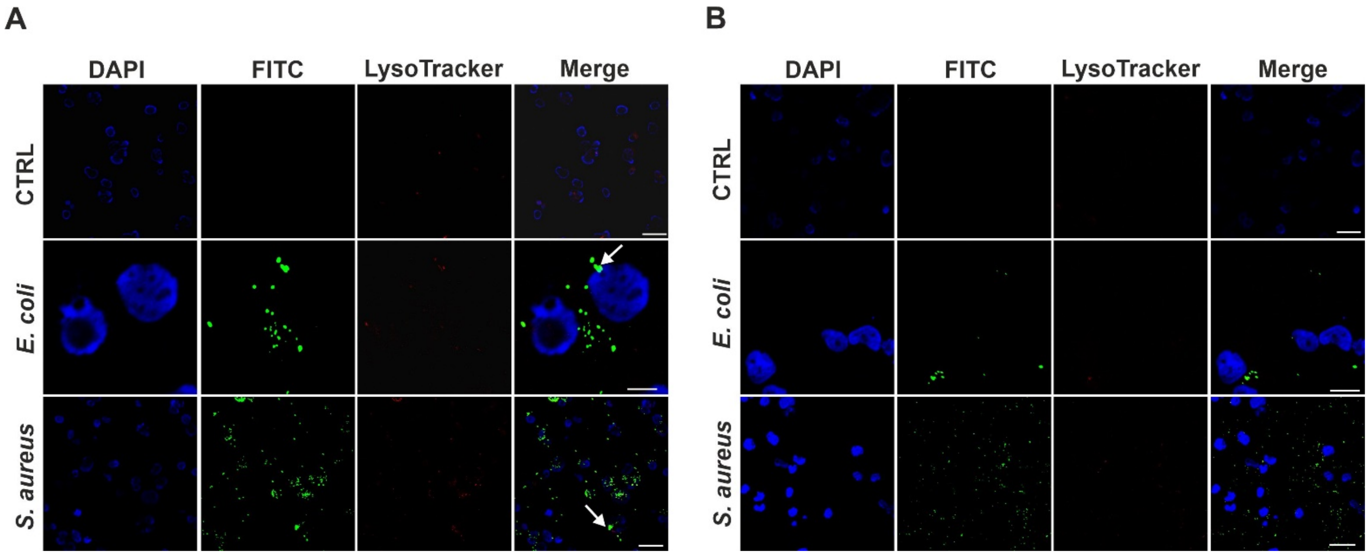

Supplementary Figure 4. Representative CLSM images of diff. THP-1 cells exposed to FITC-conjugated *E. coli* or *S. aureus* bacteria (green) strains. Subsequently, LysoTracker staining was applied to detect colocalized engulfed bacteria with lysosomes (red) in diff. THP-1 cells. Colocalization can be observed in diff. THP-1 cells without inhibitors (**A**, arrows), while pretreatment of 5 µM cytochalasin D (**B**) decreased the amount of active phagocytic cells. Nuclear counterstaining was performed with DAPI (blue). Scale bars: 50 µm (CTRL – **A**, **B**; *S. aureus*- **A**, **B**), 10 µm (*E. coli* - **A**), 20 µm (*E. coli* – **B**).

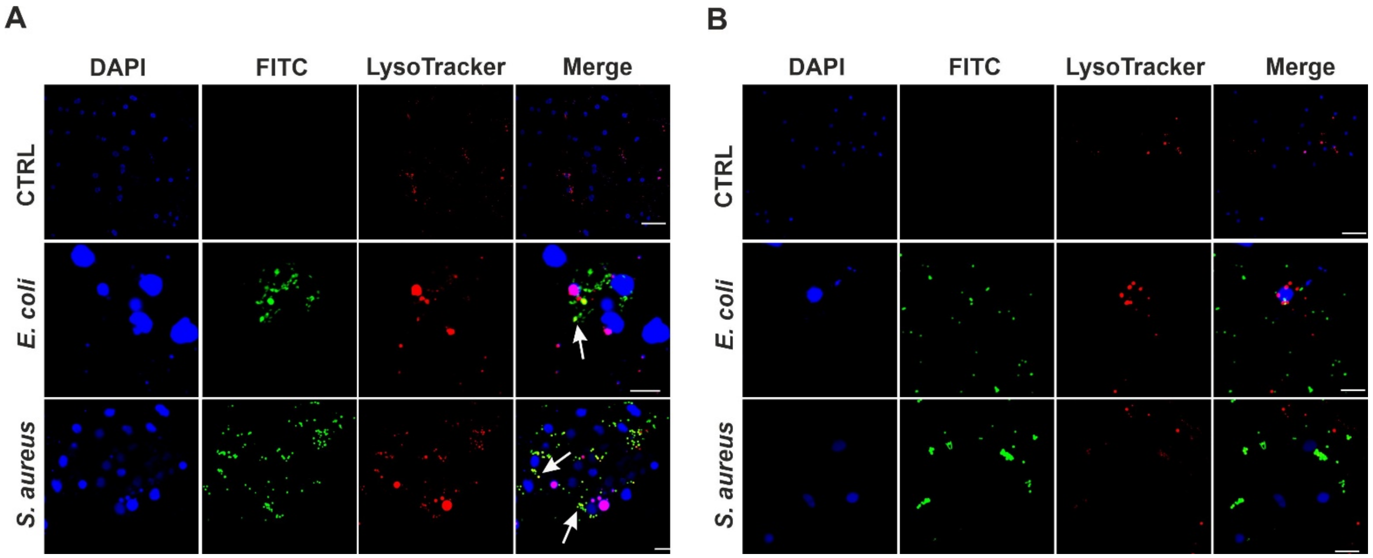

Supplementary Figure 5. Representative CLSM images of coelomocytes incubated with FITC-conjugated *E. coli* or *S. aureus* bacteria (green). LysoTracker staining was applied to visualize the co-localized engulfed bacteria with lysosomes (red) of coelomocytes (**A**, arrows), while pretreatment of 5 µM cytochalasin D (**B**) decreased the level of engulfed bacteria in coelomocytes. Nuclear counterstaining was performed with DAPI (blue). Scale bars: 50 µm (CTRL - **A**, **B**), 10 µm (*E. coli*- **A**, **B**; *S. aureus* - **A**, **B**).
